# A94 ASSOCIATION OF BOWEL URGENCY WITH OTHER DISEASE ACTIVITY MEASURES IN ULCERATIVE COLITIS

**DOI:** 10.1093/jcag/gwae059.094

**Published:** 2025-02-10

**Authors:** P Gill, H Finch, L Stein, C Ma

**Affiliations:** Medicine, University of Calgary, Calgary, AB, Canada; Medicine, University of Calgary, Calgary, AB, Canada; Medicine, University of Calgary, Calgary, AB, Canada; Medicine, University of Calgary, Calgary, AB, Canada

## Abstract

**Background:**

The presence of bowel urgency and fecal incontinence negatively impacts quality of life in patients with ulcerative colitis (UC).

**Aims:**

We aimed to characterize the assessment of urgency and incontinence in routine clinical care.

**Methods:**

We conducted a retrospective cohort study of prevalent cases of UC evaluated in the University of Calgary IBD Clinic from December 2022 to May 2023, with at least 12 months of follow-up to May 2024. Data on the evaluation of bowel urgency was collected, as were other patient-, disease-, and outcome-related parameters. We aimed to evaluate (1) the proportion of clinic visits where bowel urgency was assessed and documented; (2) the methods for evaluating urgency; (3) the correlation between bowel urgency and other patient-reported outcome measures (including stool frequency and rectal bleeding); and (4) the correlation between bowel urgency and endoscopic appearance.

**Results:**

A total of 274 patients with UC who attended 435 clinic visits were included in this analysis (126 [46.0%] male, mean age at diagnosis 33.2 years [SD 15.5], 104 [40.3%] pancolitis. At the index visit, bowel urgency was evaluated in 160/274 visits (58.4%) and was present in 24.4% (39/160). Most cases were evaluated using the presence/absence of bowel urgency, rather than using a Likert or ordinal scale. In 416 follow-up visits, bowel urgency was assessed in 231 (55.5%) of visits and was present in 75 cases (33.0%). There were moderate-to-strong correlations between bowel urgency and stool frequency (Spearman correlation coefficient 0.54, p<0.001) and rectal bleeding (0.53, p<0.001), but only weak correlation with endoscopic evaluation (Spearman 0.20, p=0.08). Among 136 patients with normal or near normal stool frequency, 16.2% (22/136) had persistent bowel urgency. Among 59 patients with normal or near normal endoscopy, 20.3% (12/59) had persistent bowel urgency.

**Conclusions:**

Bowel urgency remains inconsistently evaluated in clinical care, representing an important area for quality improvement. Although bowel urgency was significantly associated with other patient-reported outcome measures, approximately 1 in 5 patients will have persistent bowel urgency despite minimal endoscopic activity.

**Funding Agencies:**

None

